# Apoptotic Cell Clearance and Its Role in the Origin and Resolution of Chronic Inflammation

**DOI:** 10.3389/fimmu.2015.00139

**Published:** 2015-03-25

**Authors:** Luis Enrique Muñoz, Christian Berens, Kirsten Lauber, Udo S. Gaipl, Martin Herrmann

**Affiliations:** ^1^Department of Internal Medicine 3, Rheumatology and Immunology, Friedrich-Alexander University of Erlangen-Nürnberg, Erlangen, Germany; ^2^Institute of Molecular Pathogenesis, Friedrich-Loeffler-Institut, Federal Research Institute for Animal Health, Jena, Germany; ^3^Department of Radiation Oncology, Ludwig-Maximilians-University Munich, Munich, Germany; ^4^Department of Radiation Oncology, University Hospital Erlangen, Friedrich-Alexander University of Erlangen-Nürnberg, Erlangen, Germany

**Keywords:** clearance, cell death, phagocytosis, chronic, inflammation, cancer, atherosclerosis, autoimmunity

During normal tissue turnover, innate immune sentinels swiftly clear dying cells in an immunologically silent manner. Large amounts of nuclear chromatin are meticulously kept away from the immune system to prevent inflammation and, eventually, autoimmunity from developing. In case this highly efficient surveillance system is derailed, the unanticipated presence of *post-mortem* remnants in tissues can challenge the otherwise normally ensuing immunological tolerance. Apoptotic cell death is the most natural way to preserve this precious tissue homeostasis. Early recognition and swift clearance of cells undergoing apoptosis ensures the prevention of tissue damage and autoimmune reactions (1). Kimani et al. thoroughly review in this Research Topic, the most recent evidence linking autoimmune diseases and the recognition of apoptotic cells via surface-exposed phosphatidylserine (2).

Besides the autoimmune phenotype of chronic inflammatory rheumatoid disorders, a plethora of pathologies have been associated with defects in genes involved in the clearance of cell remnants from tissues (3, 4). This Research Topic bundles a set of manuscripts describing various ways of how such “uncleared” cell remnants participate in the pathogenesis of chronic inflammatory diseases and also cancer. Improving our knowledge of the immune modulatory language(s) spoken by dying and dead cells and their constituents may prove essential for understanding the key processes involved. Ultimately and hopefully, this may lead to the development of new classes of therapeutic and disease-modifying agents (5).

For example, González and Hidalgo emphasize that it is now possible to take advantage of the huge amount of published evidence on therapeutic modulation of the liver X receptor activity in clearance-associated diseases (6). Notably, pharmacological regulation of such nuclear factors, which are activated upon recognition of dying cells, may enhance the ability of macrophages to clear dead cells and thereby provide additional beneficial effects for treating clearance-related diseases like osteoporosis, rheumatoid arthritis, atherosclerosis, diabetes, and Alzheimer’s disease (7–10).

Upon recruitment to sites of acute inflammation, neutrophils respond either with phagocytosis of the inflammatory trigger, degranulation, or with the formation of neutrophil-extracellular-traps (NETs) (11) exposing modified chromatin at the site of the initial injury (12). The nature of this material implies the massive death of neutrophils, and this response is important for both the inactivation of the aggressor and the resolution of the initial inflammation (13). However, how this battlefield is finally cleaned up and cleared has not been studied yet and may surely provide new therapeutic options for autoimmune diseases (12, 14). Intense current research on the immunobiology of this special way of dying called NETosis also promises new therapeutic targets for ameliorating autoinflammation (15, 16). Severe and standard treatment resistant forms of pulmonary inflammation may also profit by novel dual interventions targeting both cell survival and promotion of apoptotic cell clearance by phagocytes as Felton et al. and Szondy et al. summarize in this issue (17, 18). Although one of the multiple mechanisms of action of glucocorticoids is to enhance apoptotic cell clearance by macrophages (19), their long-term use has many side effects that strongly burden chronically diseased patients leading to higher rates of morbidity. Alternatives to classic therapies and specific pathogenesis-targeted therapies are therefore very much welcome.

The many different signals expressed or secreted by apoptotic cells noticeably determine the reaction of the organism to the event triggering death. Any shortcomings in phagocytic clearance, either by impaired clearance, excessive death or any other reason, are invariably related to continuous stimulation of the organism by either pro-inflammatory/destructive or anti-inflammatory/healing signaling. Many chronic inflammatory diseases are driven and somehow modulated by metabolites released from dying cells. For example, Chen et al. present an overview of the various sites of action of nucleotides in inflammatory conditions (20). Intervention at this level may shift the balance toward anti-inflammation, thereby achieving the therapeutic goal more effectively.

In the case of solid tumors, the metabolites and, especially, the danger signals released may serve as biomarkers. Gehrmann et al. nicely demonstrate in this Research Topic that stress response proteins are released already by premalignant conditions of the liver as well as by hepatocellular carcinoma (21). This can be important for prognosis, prediction, and monitoring. The tumor microenvironment, especially directly after anti-cancer treatment, is overflowing with mediators and signals from dead and dying cells. To obtain an efficient anti-tumor immune response, an immune-suppressive microenvironment has to be shifted to an activating one. The latter might be achieved by rendering the tumor cells immunogenic, namely, by inducing immunogenic tumor cell death forms by standard treatments such as radio- and/or chemotherapy (22). Furthermore, short range danger signals foster leukocyte infiltration into the tumor and initiate an inflammatory response, which is afterwards supplanted by long-range healing and regenerative signals, which then, in contrast, may support tumor proliferation. The review from Willems summarizes evidence documenting the dark side of apoptosis in modulating anti-tumor responses (23). A delicate balance exists between anti-tumor reactions and counteracting immune suppression. In this scenario, the role of tumor associated macrophages as sensors and central orchestrators of tumor-promoting reparatory and anti-inflammatory signals has recently been highlighted by Ford et al. (24). In addition, avoiding tumor repopulation after anti-cancer therapy by considering the immune-suppressive consequences of apoptotic cell clearance should be taken into account as a cautionary premise for each and every anti-cancer treatment (25–28). Of note, inflammatory reactions, DNA damage responses, and cell death forms are highly interconnected (29). Alterations in the clearance of dying and dead cells, their remnants, and their constituents that leak out after membrane rupture are therefore central elements in all inflammatory conditions, starting from its origin and ending in its resolution.

## Conflict of Interest Statement

The authors declare that the research was conducted in the absence of any commercial or financial relationships that could be construed as a potential conflict of interest.
